# Outcomes Following Single-Level Spinal Fusion in Patients With Systemic Lupus Erythematosus

**DOI:** 10.7759/cureus.115736

**Published:** 2026-09-03

**Authors:** Patrizia M Manziano, Grace Wilsey, Sarah Kebaish, Ved Vengsarkar, Joyce En-Hua Wang, Madison Sroufe, Kara McConaghy, Wendy Novicoff, Xudong Li, Stephen D Lockey

**Affiliations:** 1 Department of Orthopaedic Surgery, University of Virginia School of Medicine, Charlottesville, USA; 2 Department of Orthopaedic Surgery, Rutgers New Jersey Medical School, Newark, USA; 3 Department of Orthopaedic Surgery, University of Virginia Health System, Charlottesville, USA

**Keywords:** cervical fusion, healthcare utilization, immunomodulatory therapy, lumbar fusion, postoperative outcomes, systemic lupus erythematosus

## Abstract

Introduction

Patients with systemic lupus erythematosus (SLE) experience an increased risk of complications and healthcare expenditures after surgery, yet there is limited knowledge regarding outcomes after spinal fusion. This study aimed to investigate outcomes after single-level cervical and lumbar fusion in patients with SLE. A secondary objective was to assess the effect of immunomodulatory therapy (IMT) within the SLE cohort on lumbar fusion outcomes.

Methods

In this retrospective cohort study, PearlDiver was used to identify patients older than 18 years who underwent single-level anterior cervical discectomy and fusion (ACDF), posterior cervical fusion (PCF), or lumbar fusion between 2010 and 2022. Patients were assessed for age, sex, and Elixhauser Comorbidity Index and were matched in a 1:4 ratio based on SLE status. Medical and surgical complications, postoperative healthcare utilization, two-year surgical outcomes, and mortality rates were evaluated. A subgroup analysis was performed in patients with SLE with and without IMT within 90 days of lumbar fusion.

Results

After single-level cervical or lumbar fusion, patients with SLE had significantly higher rates of 90-day medical and surgical complications and healthcare utilization than their non-SLE counterparts. Following lumbar fusion, patients with SLE also had higher two-year revision rates. In the lumbar fusion subgroup analysis, patients with SLE on IMT had higher rates of cerebrovascular accidents (OR 1.87, p = 0.020), atelectasis (OR 1.75, p = 0.006), 30-day ED visits (OR 2.44, p < 0.001), 90-day ED visits (OR 1.34, p < 0.001), and 30-day readmissions (OR 1.57, p < 0.001).

Conclusion

SLE was associated with higher odds of several postoperative complications and healthcare utilization outcomes after single-level cervical or lumbar fusion, as well as higher two-year revision rates following lumbar fusion. These findings suggest that SLE may represent an important marker of perioperative risk. Additional research is needed to identify mitigation strategies and better understand the perioperative risks associated with IMT in this population.

## Introduction

Systemic lupus erythematosus (SLE) is a chronic autoimmune disorder marked by autoantibody production, resulting in aberrant inflammation that impacts multiple organ systems [1]. Patients with SLE are at increased risk of diminished bone quality compared with the general population due to systemic, serological, metabolic, hormonal, and possibly genetic contributors [2]. Li EK et al. found that patients with lupus have a higher prevalence of vertebral fractures and lower bone mineral density (BMD) compared with healthy controls, with 60% of participants with SLE having osteopenia in the lumbar spine [3]. Ruaro B et al. demonstrated that patients with SLE had significantly lower lumbar spine trabecular bone scores and BMD compared with controls [4]. Another study found a high prevalence of vertebral deformities in premenopausal patients with SLE despite normal BMD in some cases, indicating that density scores may not always correlate with bone health within the spinal column in this population [5].

Bone health and healing potential in patients with SLE may also be impacted by medications used to manage the condition. Immunosuppressive agents impede wound healing and increase the risk of postoperative infection [6]. Corticosteroids are known to impact bone density, with one study finding a dose-dependent relationship between glucocorticoid use and spinal bone loss in patients with SLE [1,2]. In addition, an observational study reported an association between a history of hydroxychloroquine treatment and lower spinal BMD compared with patients without such treatment [7]. Given these concerns regarding bone quality, surgery involving instrumentation, such as cervical or lumbar fusion, may present considerable challenges in patients with SLE.

Prior studies have shown that patients with autoimmune conditions experience higher complication rates following surgical correction of spinal deformity [8]. A retrospective investigation of lumbar fusion outcomes in patients with rheumatoid arthritis (RA) identified trends toward increased wound- and implant-related complications [9]. A recent analysis found that patients with SLE who underwent single-level posterior lumbar fusion experienced higher rates of 90-day postoperative complications [10]. Another investigation in patients with SLE found significantly higher risks of postoperative mortality and major complications, including acute renal failure, pneumonia, and septicemia, compared with patients without the condition [11]. Other studies have reported that patients with SLE experience higher rates of perioperative complications and hospital-related expenditures after orthopaedic surgery, including knee and hip arthroplasties, compared with non-SLE counterparts [12,13,14].

Given the limited literature evaluating procedure-specific outcomes following spinal fusion in patients with SLE, the purpose of this investigation was to evaluate the rates of postoperative complications and healthcare utilization in patients with SLE who underwent single-level cervical and lumbar fusion. The central hypothesis was that patients with SLE would experience higher rates of medical and operative complications compared with matched controls. As a secondary objective, a subgroup analysis was conducted to assess the effect of immunomodulatory therapy (IMT) on lumbar fusion outcomes in patients with SLE.

## Materials and methods

Data source

The data used in this analysis were obtained from the multipayer database PearlDiver (PearlDiver Inc., Colorado Springs, Colorado, USA), which contains records for over 161 million patients and includes information encoded using International Classification of Diseases (ICD) and Current Procedural Terminology (CPT) codes. All records are deidentified and compliant with the Health Insurance Portability and Accountability Act (HIPAA). Accordingly, this study was exempt from Institutional Review Board review and approval.

Study populations

For the cervical fusion study, adult patients who underwent single-level anterior cervical discectomy and fusion (ACDF) (CPT-22551, CPT-22554) or single-level posterior cervical fusion (PCF) (CPT-22600) were identified from 2010 to 2022. For the lumbar fusion study, adult patients who underwent single-level lumbar fusion were identified from 2010 to 2022 (CPT-22633, CPT-22558, CPT-22612, CPT-22630). Patients with multilevel procedures on the same day or with recent trauma or infectious indications within the 90 days prior to surgery were also excluded. Additionally, ACDF patients who underwent same-day PCF and PCF patients who underwent same-day ACDF were excluded. Two groups were generated for each procedure based on a prior diagnosis of SLE, defined by ICD-9/10 codes. Patients were assessed for age, sex, and Elixhauser Comorbidity Index (ECI) and were matched in a 1:4 ratio using exact matching to control for potential differences in baseline characteristics. In the ACDF cohort, 2,458 patients with SLE were matched with 9,819 controls; in the PCF cohort, 127 patients with SLE were matched with 501 controls; and in the lumbar fusion cohort, 3,816 patients with SLE were matched with 15,245 controls.

Outcomes

Ninety-day medical and surgical complications were evaluated, including myocardial infarction, arrhythmia, cerebrovascular accident (CVA), respiratory failure, atelectasis, pulmonary embolism (PE), pleural effusion, UTI, urinary retention, renal failure, sepsis, spinal cord deficit, cervical radiculopathy, and nerve root sensory and motor deficits. Two-year outcomes, including pseudoarthrosis and mortality, were also assessed, with revision additionally evaluated following lumbar fusion. Healthcare utilization outcomes included 30- and 90-day ED visits and inpatient readmissions. PearlDiver does not receive specific mortality data; therefore, mortality was defined using sudden/unexpected death and hospice treatment codes to capture patients who had entered end-of-life care as a proxy. For lumbar fusion, revision was defined as reinsertion of spinal fixation devices, removal or addition of spinal instrumentation, or any subsequent exploration of the fusion.

Statistical analysis

Pearson’s χ² test with Yates’ continuity correction and Welch’s t-test were used to evaluate demographic characteristics and comorbidities. Degrees of freedom were reported for all Pearson χ² tests. To complement statistical significance in the setting of large sample sizes, Cramér’s V was calculated as an effect-size measure for categorical comparisons using (V=\sqrt{\frac{\chi^{2}}{N\times \min(r-1, c-1)}}), where χ² is the Pearson chi-square statistic, N is the total sample size for the comparison, and r and c are the numbers of rows and columns in the contingency table. Because all categorical outcome comparisons were 2 × 2 tables, the equation simplified to (V=\sqrt{\frac{\chi^{2}}{N}}). Univariate analysis was initially performed to identify significant differences in postoperative outcomes. A multivariable analysis was performed using previously identified significant variables after controlling for age, sex, and ECI. All statistical analyses were conducted using the interface provided by PearlDiver Bellwether, with Cramér’s V calculated from the PearlDiver-generated Pearson χ² statistics and contingency-table sample sizes. Statistical significance was determined at p < 0.05. Given the exploratory nature of the analysis, no formal adjustment for multiple comparisons was applied.

Subgroup analysis in lumbar fusion

A subgroup analysis was performed for the lumbar fusion study by identifying patients with SLE who had at least one qualifying IMT prescription claim during the 90 days preceding the index lumbar fusion. IMT medications of interest included corticosteroids (hydrocortisone, methylprednisolone, dexamethasone), hydroxychloroquine, and biologics (belimumab, rituximab). Prescription claims were used as a proxy for medication exposure; the database could not establish adherence, treatment duration, or whether medication use continuously overlapped with the operative period. Postoperative outcomes, as described previously, were assessed for each cohort.

## Results

Cervical fusion

The ACDF cohort included 2,458 patients with SLE and 9,819 matched patients without SLE, while the PCF cohort included 127 patients with SLE and 501 matched patients without SLE. All percentages represent unadjusted event rates, whereas the reported p-values were derived from multivariable analyses. Compared with matched non-SLE controls, patients with SLE undergoing single-level ACDF had significantly higher odds of multiple 90-day medical complications, including cardiovascular, thromboembolic, genitourinary, renal, and infectious complications (Table 1). Among the most notable differences in unadjusted event rates were arrhythmia (15.1% vs. 5.9%), UTI (18.8% vs. 4.8%), and cervical radiculopathy (36.2% vs. 17.8%), with all three outcomes remaining significant on multivariable analysis (p < 0.001).

**Table 1:** Univariate and multivariable analysis of 90-day medical and surgical complications, two-year pseudoarthrosis, 30- and 90-day healthcare utilization, and two-year mortality in both groups undergoing single-level ACDF. SLE: Systemic lupus erythematosus; ACDF: Anterior cervical discectomy and fusion; CVA: Cerebrovascular accident; PE: Pulmonary embolism. Pearson χ² values were calculated using Yates’ continuity correction. Cramér’s V was calculated from the corresponding Pearson χ² statistic.

Outcome	SLE (N = 2,458), n	SLE, %	No SLE (N = 9,819), n	No SLE, %	Pearson χ² (df)	Univariate p-value	Cramér’s V	Multivariable adjusted OR (95% CI)	Multivariable p-value
90-day medical complications									
Arrhythmia	371	15.1	583	5.9	228.68 (1)	<0.001	0.136	3.07 (2.65-3.55)	<0.001
Myocardial infarction	22	0.9	25	0.3	19.50 (1)	0.002	0.04	3.57 (1.99-6.37)	0.001
CVA	68	2.8	66	0.7	77.94 (1)	<0.001	0.08	4.38 (3.09-6.22)	<0.001
Atelectasis	98	4	147	1.5	61.05 (1)	<0.001	0.071	2.77 (2.13-3.60)	<0.001
Respiratory failure	53	2.2	158	1.6	3.17 (1)	0.075	0.016	-	-
Pleural effusion	47	1.9	75	0.8	25.19 (1)	<0.001	0.045	2.56 (1.76-3.69)	<0.001
PE	29	1.2	25	0.3	36.34 (1)	<0.001	0.054	4.71 (2.75-8.13)	<0.001
Urinary retention	65	2.6	109	1.1	32.03 (1)	<0.001	0.051	2.44 (1.78-3.33)	<0.001
UTI	461	18.8	474	4.8	540.02 (1)	<0.001	0.21	4.85 (4.21-5.58)	<0.001
Renal failure	93	3.8	162	1.6	42.96 (1)	<0.001	0.059	2.47 (1.88-3.23)	<0.001
Sepsis	30	1.2	46	0.5	16.87 (1)	<0.001	0.037	2.64 (1.64-4.18)	<0.001
Surgical complications									
Cervical radiculopathy	890	36.2	1,747	17.8	394.24 (1)	<0.001	0.179	2.64 (2.39-2.91)	<0.001
Spinal cord deficit	19	0.8	118	1.2	1.75 (1)	0.187	0.012	-	-
Pseudoarthrosis (2 years)	109	4.4	239	2.4	27.84 (1)	<0.001	0.048	1.86 (1.47-2.34)	<0.001
Healthcare utilization and mortality									
ED visit (30 days)	648	26.4	921	9.4	507.16 (1)	<0.001	0.203	3.68 (3.27-4.13)	<0.001
ED visit (90 days)	945	38.4	1,631	16.6	564.00 (1)	<0.001	0.214	3.39 (3.06-3.75)	<0.001
Inpatient readmission (30 days)	334	13.6	350	3.6	373.55 (1)	<0.001	0.174	4.45 (3.79-5.22)	<0.001
Inpatient readmission (90 days)	509	20.7	517	5.3	610.11 (1)	<0.001	0.223	5.02 (4.38-5.75)	<0.001
Mortality (2 years)	1	0	15	0.2	1.13 (1)	0.287	0.01	-	-

Likewise, patients with SLE undergoing single-level PCF had significantly higher odds of several postoperative complications, including arrhythmia, myocardial infarction, CVA, UTI, renal failure, and cervical radiculopathy (Table 2). Within two years postoperatively, pseudoarthrosis was more common among patients with SLE after both ACDF (4.4% vs. 2.4%) and PCF (18.9% vs. 10.6%); both differences remained significant on multivariable analysis. Patients with SLE also had higher rates of ED visits and inpatient readmissions at both 30 and 90 days following cervical fusion (p < 0.001 for all) (Tables 1-2). Statistical comparisons for two-year mortality in the PCF cohort were not estimable because no mortality events occurred in either group.

**Table 2:** Univariate and multivariable analysis of 90-day medical and surgical complications, two-year pseudoarthrosis, 30- and 90-day healthcare utilization, and two-year mortality in both groups undergoing single-level PCF. SLE: Systemic lupus erythematosus; PCF: Posterior cervical fusion; CVA: Cerebrovascular accident; PE: Pulmonary embolism. Pearson χ² values were calculated using Yates’ continuity correction. Cramér’s V was calculated from the corresponding Pearson χ² statistic. For PCF mortality, both groups had zero events; therefore, χ² and Cramér’s V were not estimable.

Outcome	Systemic lupus erythematosus (N = 127), n	Systemic lupus erythematosus, %	No systemic lupus erythematosus (N = 501), n	No systemic lupus erythematosus, %	Pearson chi-square (degrees of freedom)	Univariate p-value	Cramér’s V	Multivariable adjusted OR (95% CI)	Multivariable p-value
90-day medical complications									
Arrhythmia	31	24.4	52	10.4	16.19 (1)	<0.001	0.161	2.93 (1.74-4.89)	<0.001
Myocardial infarction	4	3.1	2	0.4	3.89 (1)	0.049	0.079	5.65 (1.20-26.55)	0.028
Cerebrovascular accident	7	5.5	8	1.6	5.09 (1)	0.024	0.09	3.68 (1.22-10.92)	0.018
Atelectasis	14	11	29	5.8	3.57 (1)	0.059	0.075	-	-
Respiratory failure	9	7.1	23	4.6	0.84 (1)	0.359	0.037	-	-
Pleural effusion	6	4.7	19	3.8	0.05 (1)	0.821	0.009	-	-
Pulmonary embolism	2	1.6	2	0.4	0.74 (1)	0.388	0.034	-	-
Urinary retention	3	2.4	19	3.8	0.26 (1)	0.608	0.02	-	-
UTI	32	25.2	38	7.6	29.98 (1)	<0.001	0.218	4.46 (2.59-7.68)	<0.001
Renal failure	11	8.7	17	3.4	5.42 (1)	0.02	0.093	3.21 (1.32-7.64)	0.009
Sepsis	3	2.4	8	1.6	0.04 (1)	0.835	0.008	-	-
Surgical complications									
Cervical radiculopathy	42	33.1	80	16	17.86 (1)	<0.001	0.169	2.67 (1.70-4.17)	<0.001
Spinal cord deficit	8	6.3	20	4	0.78 (1)	0.376	0.035	-	-
Pseudoarthrosis (2 years)	24	18.9	53	10.6	5.77 (1)	0.016	0.096	2.08 (1.19-3.58)	0.009
Healthcare utilization and mortality									
ED visit (30 days)	35	27.6	49	9.8	26.13 (1)	<0.001	0.204	3.67 (2.22-6.07)	<0.001
ED visit (90 days)	51	40.2	96	19.2	23.75 (1)	<0.001	0.194	3.07 (1.97-4.77)	<0.001
Inpatient readmission (30 days)	31	24.4	45	9	21.24 (1)	<0.001	0.184	3.45 (2.03-5.83)	<0.001
Inpatient readmission (90 days)	43	33.9	57	11.4	36.59 (1)	<0.001	0.241	4.37 (2.70-7.10)	<0.001
Mortality (2 years)	0	0	0	0	Not estimable	NA	Not estimable	-	-

Lumbar fusion

The lumbar fusion cohort included 3,816 patients with SLE and 15,245 matched patients without SLE. All percentages represent unadjusted event rates, whereas the reported p-values were derived from multivariable analyses. Compared with matched controls, patients with SLE had significantly higher adjusted odds of multiple 90-day medical complications after single-level lumbar fusion, including cardiovascular, pulmonary, thromboembolic, genitourinary, renal, and infectious complications (Table 3). Among the largest differences in unadjusted event rates were UTI (21.4% vs. 6.1%) and arrhythmia (17.2% vs. 7.8%). Patients with SLE also experienced higher rates of pseudoarthrosis (4.9% vs. 2.9%) and revision surgery (7.5% vs. 3.2%) within two years. Healthcare utilization was similarly greater, with higher rates of 30- and 90-day ED visits and inpatient readmissions (Table 3).

**Table 3:** Univariate and multivariable analysis of 90-day medical and surgical complications, two-year revision rates, 30- and 90-day healthcare utilization, and two-year mortality in both groups undergoing single-level lumbar fusion. SLE: Systemic lupus erythematosus; CVA: Cerebrovascular accident; PE: Pulmonary embolism. Pearson χ² values were calculated using Yates’ continuity correction. Cramér’s V was calculated from the corresponding Pearson χ² statistic.

Outcome	Systemic lupus erythematosus (N = 3,816), n	Systemic lupus erythematosus, %	No systemic lupus erythematosus (N = 15,245), n	No systemic lupus erythematosus, %	Pearson chi-square (degrees of freedom)	Univariate p-value	Cramér’s V	Multivariable adjusted OR (95% CI)	Multivariable p-value
90-day medical complications									
Arrhythmia	655	17.2	1,189	7.8	305.27 (1)	<0.001	0.127	2.56 (2.30-2.85)	<0.001
Myocardial infarction	50	1.3	71	0.5	33.19 (1)	<0.001	0.042	2.86 (1.98-4.12)	<0.001
Cerebrovascular accident	100	2.6	106	0.7	104.02 (1)	<0.001	0.074	3.94 (2.98-5.21)	<0.001
Atelectasis	193	5.1	269	1.8	138.56 (1)	<0.001	0.085	3.01 (2.48-3.64)	<0.001
Respiratory failure	102	2.7	220	1.4	27.06 (1)	<0.001	0.038	1.89 (1.48-2.40)	<0.001
Pleural effusion	116	3	120	0.8	124.82 (1)	<0.001	0.081	3.98 (3.07-5.16)	<0.001
Pulmonary embolism	49	1.3	58	0.4	43.04 (1)	<0.001	0.048	3.41 (2.32-5.00)	<0.001
Urinary retention	126	3.3	298	2	24.85 (1)	<0.001	0.036	1.72 (1.39-2.12)	<0.001
UTI	817	21.4	931	6.1	856.22 (1)	<0.001	0.212	4.40 (3.97-4.89)	<0.001
Renal failure	204	5.3	350	2.3	99.54 (1)	<0.001	0.072	2.49 (2.07-2.98)	<0.001
Sepsis	59	1.5	105	0.7	25.31 (1)	<0.001	0.036	2.27 (1.63-3.12)	<0.001
Surgical complications									
Nerve root sensory deficit	45	1.2	155	1	0.63 (1)	0.428	0.006	-	-
Nerve root motor deficit	126	3.3	589	3.9	2.51 (1)	0.113	0.011	-	-
Pseudoarthrosis (2 years)	186	4.9	435	2.9	38.91 (1)	<0.001	0.045	1.75 (1.46-2.08)	<0.001
Revision (2 years)	286	7.5	488	3.2	143.33 (1)	<0.001	0.087	2.45 (2.11-2.85)	<0.001
Healthcare utilization and mortality									
ED visit (30 days)	958	25.1	1,448	9.5	672.58 (1)	<0.001	0.188	3.37 (3.07-3.70)	<0.001
ED visit (90 days)	1,384	36.3	2,454	16.1	770.97 (1)	<0.001	0.201	3.18 (2.93-3.45)	<0.001
Inpatient readmission (30 days)	706	18.5	1,023	6.7	512.99 (1)	<0.001	0.164	3.24 (2.91-3.60)	<0.001
Inpatient readmission (90 days)	1,002	26.3	1,319	8.7	882.99 (1)	<0.001	0.215	3.91 (3.56-4.29)	<0.001
Mortality (2 years)	5	0.1	9	0.1	1.29 (1)	0.257	0.008	-	-

In the subgroup analysis, 2,478 patients with SLE were prescribed IMT and 1,338 were not within 90 days preoperatively. Patients with SLE on IMT were found to have higher rates of CVA (2.6% vs. 1.4%, p = 0.020) and atelectasis (4.4% vs. 2.5%, p = 0.006) following lumbar fusion compared with patients not on IMT. Interestingly, there were no significant differences in the rates of pseudoarthrosis and revision surgery at two years between patients with SLE who were on IMT and those who were not. Multivariable analysis also demonstrated that patients with SLE on IMT experienced higher rates of ED visits at 30 and 90 days postoperatively, as well as higher rates of readmission at 30 days postoperatively (p < 0.001 for all) (Table 4).

**Table 4:** Univariate and multivariable analysis of 90-day medical and surgical complications, two-year revision rates, 30- and 90-day healthcare utilization, and two-year mortality in patients with SLE undergoing single-level lumbar fusion, stratified by IMT use. SLE: Systemic lupus erythematosus; IMT: Immunomodulatory therapy; CVA: Cerebrovascular accident; PE: Pulmonary embolism. Pearson χ² values were calculated using Yates’ continuity correction. Cramér’s V was calculated from the corresponding Pearson χ² statistic.

Outcome	SLE with IMT (N = 2,478), n	SLE with IMT, %	SLE without IMT (N = 1,338), n	SLE without IMT, %	Pearson chi-square (degrees of freedom)	Univariate p-value	Cramér’s V	Multivariable adjusted OR (95% CI)	Multivariable p-value
90-day medical complications									
Arrhythmia	350	14.1	178	13.3	0.42 (1)	0.515	0.011	-	-
Myocardial infarction	30	1.2	9	0.7	1.98 (1)	0.159	0.023	-	-
Cerebrovascular accident	64	2.6	19	1.4	4.99 (1)	0.026	0.036	1.87 (1.13-3.26)	0.02
Atelectasis	109	4.4	34	2.5	7.81 (1)	0.005	0.045	1.75 (1.19-2.64)	0.006
Respiratory failure	55	2.2	21	1.6	1.56 (1)	0.211	0.02	-	-
Pleural effusion	60	2.4	30	2.2	0.06 (1)	0.813	0.004	-	-
Pulmonary embolism	24	1	16	1.2	0.24 (1)	0.623	0.008	-	-
Urinary retention	63	2.5	35	2.6	<0.001 (1)	0.976	0	-	-
UTI	438	17.7	211	15.8	2.10 (1)	0.147	0.023	-	-
Renal failure	110	4.4	53	4	0.38 (1)	0.54	0.01	-	-
Sepsis	35	1.4	11	0.8	2.07 (1)	0.15	0.023	-	-
Surgical complications									
Nerve root sensory deficit	38	1.5	18	1.3	0.10 (1)	0.749	0.005	-	-
Nerve root motor deficit	78	3.1	40	3	0.03 (1)	0.864	0.003	-	-
Pseudoarthrosis (2 years)	129	5.2	57	4.3	1.48 (1)	0.224	0.02	-	-
Revision (2 years)	191	7.7	95	7.1	0.38 (1)	0.538	0.01	-	-
Healthcare utilization and mortality									
ED visit (30 days)	742	29.9	216	16.1	87.27 (1)	<0.001	0.151	2.44 (2.04-2.92)	<0.001
ED visit (90 days)	792	32	355	26.5	11.93 (1)	<0.001	0.056	1.34 (1.15-1.58)	<0.001
Inpatient readmission (30 days)	512	20.7	194	14.5	21.48 (1)	<0.001	0.075	1.57 (1.30-1.89)	<0.001
Inpatient readmission (90 days)	532	21.5	275	20.6	0.38 (1)	0.536	0.01	-	-
Mortality (2 years)	3	0.1	2	0.1	<0.001 (1)	1	0	-	-

Cramér’s V values were generally small across the univariate comparisons (V = 0.001-0.215).

## Discussion

Patients with autoimmune diseases are known to face an elevated risk of complications and postoperative mortality after spine surgery and other major surgical procedures [8,9,11]. This study demonstrated that SLE is associated with higher rates of medical and surgical complications and healthcare utilization following spinal fusion, as well as higher revision rates following lumbar fusion. These findings align with prior evidence showing that patients with SLE have higher rates of postoperative complications [11].

The combination of chronic inflammation and organ damage in SLE, together with the physiologic stress of surgery, may increase the risk of postoperative medical complications in these patients. Lupus is a known risk factor for cardiovascular events in the postoperative period [15]. One report investigating perioperative major adverse cardiovascular events found that patients with SLE had a higher frequency of myocardial infarction and stroke after surgery [16]. This finding may be due to the hypercoagulable state and chronic inflammation associated with SLE, which can damage endothelial cells and trigger the coagulation cascade, leading to increased clot formation [17]. Existing literature also describes higher rates of pulmonary complications after surgery, including pneumonia, pulmonary embolism, and mechanical ventilation, in patients with lupus [11,15]. Aberrant inflammation can also affect the lungs, resulting in pleuritis, interstitial lung disease, and pulmonary hypertension, potentially predisposing patients to the postoperative complications observed in this study [18]. Additionally, lupus is known to affect the genitourinary system and kidney function [15,19]. The higher rates of renal failure observed in this study may be related to subclinical damage from autoantibody deposition in the kidneys. These complications highlight the need for careful optimization and postoperative monitoring. Consultation with medical comanagement services may prove beneficial for the early detection and management of these challenges.

Patients with SLE are known to experience higher rates of healthcare utilization after surgery. Yazdany J et al. demonstrated that patients with SLE had among the highest 30-day hospital readmission rates of any chronic condition [20]. Similarly, a nationally representative study of 132,400 hospitalized patients with SLE found a 14% 30-day readmission rate, with an increased risk among younger patients, those insured by Medicaid or Medicare, and those with severe systemic manifestations [21]. Another cohort study of Medicare beneficiaries reported that young adults with SLE had a 40% higher risk of all-cause 30-day hospital readmission compared with those without the condition [22]. These findings could be attributed to a complex interplay among disease severity, including pre-existing comorbidities and more aggressive disease in younger patients, and access to healthcare (i.e., public insurance), which may be major contributing factors to readmissions in patients with SLE. In terms of ED utilization, a systematic review by Lin DH et al. found that 25-49% of patients with SLE had at least one ED visit per year, compared with 18-35% in the general population [23]. Not only do patients with SLE appear to have higher rates of ED visits, but Dhital R et al. also reported that the disease was associated with twice the rate of inpatient admission compared with matched controls [24]. These findings illustrate the elevated healthcare utilization and costs associated with caring for patients with SLE, which are often driven by characteristic periods of disease flare-ups, predisposing comorbidities, and medication-related complications that can lead to infections and delayed wound healing.

Regarding the cervical fusion study, patients with SLE undergoing ACDF developed more complications compared with those who underwent PCF. This finding stands in contrast to a PearlDiver analysis of Medicare beneficiaries, in which one- to two-level PCF was associated with higher complication rates, except for dysphagia, compared with one- to two-level ACDF [25].

In the lumbar fusion study, patients with SLE experienced higher rates of multiple postoperative complications, healthcare utilization, pseudoarthrosis, and revision compared with matched controls. Notably, the unadjusted two-year revision rate was more than twice as high in patients with SLE (7.5% vs. 3.2%). These findings are consistent with prior database studies demonstrating increased perioperative complications after lumbar fusion in patients with SLE and other autoimmune conditions. For example, Dhodapkar MM et al. reported increased odds of several 90-day adverse events after single-level posterior lumbar fusion in patients with SLE, while Urakawa H et al. identified RA and other collagen vascular diseases as independent risk factors for subsequent lumbar fusion [9,10]. These findings may reflect, in part, the impaired bone health and increased fracture susceptibility previously described in patients with SLE [26].

Subgroup analysis within the lumbar fusion cohort demonstrated that patients with SLE with documented IMT exposure had higher adjusted odds of select medical complications and short-term healthcare utilization. Lin JA et al. previously found that patients with SLE undergoing major surgery, defined as procedures requiring general, epidural, or spinal anesthesia and at least one day of hospitalization, had an increased risk of postoperative complications and mortality, with a dose-dependent relationship between preoperative steroid use and adverse outcomes [11]. Salib A et al. found that patients with SLE on IMT had higher odds of adverse events following total shoulder arthroplasty [27]. Interestingly, although some studies have identified long-term steroid use as a significant risk factor for pseudoarthrosis in lumbar fusion surgeries in the general population, the results of this investigation found no significant difference in rates of nonunion among those on IMT [28]. However, the analysis grouped various immunomodulatory therapies, including corticosteroids, hydroxychloroquine, and biologic agents, which differ substantially in their mechanisms of action and potential effects on bone metabolism, immune function, and postoperative infection risk. Combining these medications into a single exposure category may therefore have attenuated, amplified, or otherwise obscured medication-specific associations. In addition, the analysis could not account for medication dosage, treatment duration, or concurrent use of multiple agents. Further studies examining individual medication classes are necessary to elucidate the relationship between IMT and postoperative complications and optimize perioperative planning.

Cramér’s V values provided additional context for the statistically significant univariate comparisons. Most associations between SLE status and postoperative outcomes were small in magnitude (V < 0.30), despite many highly significant p-values, particularly in the larger ACDF and lumbar fusion cohorts. The strongest associations included 90-day inpatient readmission after lumbar fusion (V = 0.215), UTI after lumbar fusion (V = 0.212), and several measures of short-term healthcare utilization after cervical and lumbar fusion (V approximately 0.18-0.22). In the IMT subgroup analysis, effect sizes were uniformly small; the largest association was observed for 30-day ED visits (V = 0.151), whereas the significant associations for CVA, atelectasis, 90-day ED visits, and 30-day readmission were smaller (V = 0.036-0.075). These findings indicate that many statistically significant associations were modest at the population level, although small effect sizes may remain clinically relevant for serious or relatively uncommon postoperative complications.

This study has several limitations. First, the analysis is limited by its dependence on administrative data and the possibility of reporting errors in medical coding. Additionally, the PearlDiver database does not represent a truly randomized sample; instead, it includes a skewed population with specific payer mixes that may over- or underrepresent certain demographic groups, which could affect the generalizability of the results. Because multiple postoperative outcomes were evaluated, the possibility of type I error cannot be excluded. The analyses should therefore be interpreted as exploratory, particularly for associations with p-values near the prespecified significance threshold. Granular clinical detail is also absent from the database, limiting the assessment of potentially important confounders, including radiographic imaging, SLE-specific factors (e.g., disease activity, organ involvement, and laboratory parameters), baseline disease severity, patient-level factors (e.g., smoking status, BMI, and osteoporosis severity), and procedure-level factors (e.g., surgical indication, surgeon experience, operative time, and blood loss). While the subgroup analysis identified patients based on prescriptions, it was not possible to determine treatment duration or patient-specific adherence. Future prospective research is necessary to explore long-term outcomes, as well as the interplay between perioperative IMT use and surgical outcomes in this vulnerable population.

## Conclusions

In this large retrospective matched-cohort study, SLE was associated with increased odds of postoperative medical complications, higher rates of pseudoarthrosis, and greater healthcare utilization following single-level cervical and lumbar fusion. Patients with SLE also had higher two-year revision rates following lumbar fusion. These findings suggest that SLE may represent an important marker of perioperative risk and support comprehensive preoperative assessment, optimization of comorbidities and bone health, and closer postoperative surveillance. Among patients with SLE undergoing lumbar fusion, IMT use was associated with higher rates of select postoperative complications and short-term healthcare utilization, although long-term surgical outcomes did not differ significantly according to IMT status. These results may support individualized patient counseling, shared surgical decision-making, and multidisciplinary perioperative management. Prospective studies incorporating disease activity, medication type, dosage, treatment duration, adherence, and perioperative management are needed to clarify these associations and identify modifiable strategies to improve outcomes in this population.
